# A Theoretical Study of the Effects of Co-Doping Ions at K and Nb Sites on the Properties of KNbO_3_ Nanoparticles

**DOI:** 10.3390/nano14181473

**Published:** 2024-09-10

**Authors:** Angel T. Apostolov, Iliana N. Apostolova, Julia M. Wesselinowa

**Affiliations:** 1 Department of Physics, Faculty of Hydrotechnics, University of Architecture, Civil Engineering and Geodesy, Hristo Smirnenski Blvd. 1, 1046 Sofia, Bulgaria; angelapos@abv.bg; 2 Faculty of Forest Industry, University of Forestry, Kl. Ohridsky Blvd. 10, 1756 Sofia, Bulgaria; inaapos@abv.bg; 3 Faculty of Physics, Sofia University “St. Kliment Ohridski”, J. Bouchier Blvd. 5, 1164 Sofia, Bulgaria

**Keywords:** co-doped KNbO_3_ nanoparticles, magnetization, polarization, band gap, microscopic model, Green’s function theory

## Abstract

Using a microscopic model and Green’s function theory, we have investigated the co-doping effect on ferroelectric KNbO_3_ nanoparticles. Let us emphasize that while the doping with transition metal ions at the Nb site leads an increase in the ferromagnetism and a reduction the band gap, it also decreases the ferroelectricity. On the other hand, doping with La or Ba at the K site leads to enhanced polarization, but does not lead to the appearance of ferromagnetism and reduction in the band gap. Therefore, we have studied co-doping with La/Cr and La/Co ions, which leads to increasing the magnetization and polarization as well as to strongly decreasing the band gap energy. Thus, we observe a multiferroic material with room-temperature ferromagnetism and ferroelectricity as well as small band gap energy which can be tuned using various co-doping ions. There is a good agreement with the existing experimental data.

## 1. Introduction

There is a large group of ferroelectric materials with a perovskite structure and general formula ${ABO}_{3}$, where *A* = Na, K, Ba, Sr, Ca, Pb, Bi, etc., and *B* = Ti, Fe, Nb, Ta, W, etc. ${\mathrm{KNbO}}_{3}$ (KNO) is a typical representative in this group, with a high Curie temperature of about 435 °C. KNO is considered a potential material for lead-free piezoelectric applications due to its high piezoelectric response and elevated Curie temperature [1,2]. At room temperature, KNO crystallizes in an orthorhombic structure [3]. In this perovskite oxide, the ferroelectric properties are derived from the displacement of ${\mathrm{Nb}}^{5+}$, which move away from the center of symmetry within the ${\mathrm{NbO}}_{6}$ octahedron, thereby breaking the spatial inversion symmetry. It is important to note that $K^{+}$ and ${\mathrm{Nb}}^{5+}$ are paramagnetic. As a result, pure bulk KNO is inherently nonmagnetic and exhibits only ferroelectric characteristics.

There are two methods for a material to become magnetic KNO, i.e., to be multiferroic, which are considered in this paper. One method is to reduce the material’s size; the other is doping with magnetic ions. Moreover, doping KNO with various ions (Co, Fe, Mn, Cr, Al) can modify all of its properties—magnetic, electric, and optical [1,4,5,6]. Thus, KNO is considered a promising candidate for multiferroic applications at room temperature. Let us emphasize that the most experimental data are for doped bulk KNO. It is difficult to synthesize ion-doped KNO nanoceramics with high quality. For example, Sun et al. have studied ${\mathrm{Er}}^{3+}$-doped KNO nanocrystals [7].

In recent years, many papers have been published which consider the co-doping effect with different ions on various properties of KNO. Multiferroic and optical properties of Ba/Ni, La/Co, and Ba/Sc that co-dope KNO at different sites have been studied by Song et al. [8], Zhang et al. [9], and Tiwari et al. [10], respectively. First-principles calculations confirm that the tuning of optical and antiferromagnetic properties in Ba/Ni co-doped KNO is due mainly to the Ni 3d electron contribution [8]. The band gap of Ba/Ni co-doped KNO is strongly reduced from 3.30 eV to 1.18 eV. In La/Co co-doped KNO, Co doping significantly reduces the bandgap, while La doping has the opposite effect [9]. When the ${\mathrm{Co}}^{2+}$ replaces part of ${\mathrm{Nb}}^{5+}$ in the KNO lattice, the ferroelectric polarization is weakened. Recent first-principles studies have indicated that Mn-doped structures, where manganese occupies both Nb and K sites, exhibit greater stability [11]. Substitution with two or more ions allows for the more effective and flexible modification of various properties of KNO required for their multiple applications.

In [12], we investigated the properties of KNO nanoparticles (NPs) through substituting transition metal ions at the Nb site or Na ions at the K site. Here, we will study co-doping effects with different ions via substitution at both the K and Nb sites on the multiferroic properties, the band gap energy of KNO NPs within a microscopic model, and Green’s function technique. The theoretical results obtained will be compared with existing experimental data.

## 2. The Model and Method

The ferroelectric properties of KNO, which arise from the displacement of ${\mathrm{Nb}}^{5+}$ ions, can be effectively modeled using the Ising model in a transverse field (TIM):

(1) $$H_{e}=-\Omega \sum_{i}B_{i}^{x}-\frac{1}{2}\sum_{ij}\left(1-x^{\prime }\right)J_{ij}^{\prime }B_{i}^{z}B_{j}^{z}.$$

In this model, $B_{i}^{x}$ and $B_{i}^{z}$ represent the pseudo-spin operators, $J_{ij}^{\prime }$ denotes the nearest-neighbor exchange pseudo-spin interaction, Ω is the tunneling frequency, and $x^{\prime }$ signifies the concentration of substituted ions at the Bi site. We adopt a new coordinate system, obtained by rotating the original system used in (1) by an angle θ in the xz plane, chosen such that $\langle B^{x^{\prime }}\rangle =0$ in this new frame. The TIM was initially proposed by Blinc and de Gennes [13] for describing order–disorder-type ferroelectrics like ${\mathrm{KH}}_{2}$${\mathrm{PO}}_{4}$ (KDP). The TIM has also been applied to displacive ferroelectrics such as ${\mathrm{BaTiO}}_{3}$ (BTO) [14].

The spontaneous polarization *P* is calculated from the Green’s function:

(2) $$G_{ij}=\ll B_{i}^{+};B_{j}^{-}\gg$$

as

(3) $$P=\frac{1}{2N^{2}}\sum_{ij}\tanh\frac{E_{fij}}{2k_{B}T}.$$

$E_{fij}$ are the excitations calculated from the following:

(4) $$E_{fij}=\frac{\langle \left[\left[B_{i}^{+},H\right],B_{j}^{-}\right]\rangle }{\langle \left[B_{i}^{+},B_{j}^{-}\right]\rangle }.$$

The magnetic properties of ion-doped KNO are modeled using a modified Heisenberg model:

$$H_{m}=-\sum_{i,j}xJ_{ij}S_{i}\cdot S_{j}-\sum_{i}D_{i}{\left(S_{i}^{z}\right)}^{2}-g\mu_{B}h\sum_{i}S_{i}^{z}.$$

In this model, $S_{i}$ represents the Heisenberg spin operator for the doping ion at site *i*. $J_{ij}$ is the exchange interaction coupling between doping ions which is dependent inversely proportional on the distance between the spins, between the lattice parameters. *x* denotes the concentration of substituted ions at the Nb site. Additionally, $D_{i}$ is the single-ion anisotropy constant, and *h* represents the external magnetic field.

The spontaneous magnetization $M=\langle S^{z}\rangle$ is calculated using the method of Tserkovnikov [15] as follows:

(5) $$M=\langle S^{z}\rangle =\frac{1}{N}\sum_{ij}\left[\left(S+0.5\right)\coth\left[\left(S+0.5\right)\beta E_{mij}\right)]-0.5\coth\left(0.5\beta E_{mij}\right)\right],$$

where *S* is the spin value, $\beta =1/k_{B}T$. $E_{mij}$ is the spin excitations calculated from the spin Green’s function $G_{ij}=\ll S_{i}^{+};S_{j}^{-}\gg$:

(6) $$E_{mij}=\frac{\langle \left[\left[S_{i}^{+},H\right],S_{j}^{-}\right]\rangle }{\langle \left[S_{i}^{+},S_{j}^{-}\right]\rangle }.$$

To determine the band gap energy of ion-doped KNO, we employ the s-d(f) model. Consequently, in addition to the Heisenberg Hamiltonian (4), we need to incorporate the following Hamiltonians: $H_{el}$ and $H_{m-el}$. $H_{el}$ represents the Hamiltonian for the conduction band electrons:

(7) $$H_{el}=\sum_{ij\sigma }t_{ij}c_{i\sigma }^{+}c_{j\sigma }+\frac{1}{2}\sum_{ijkl,\sigma \sigma^{\prime }}v\left(ijkl\right)c_{i\sigma }^{+}c_{j\sigma^{\prime }}^{+}c_{k\sigma^{\prime }}c_{l\sigma }.$$

In this context, $t_{ij}$ denotes the hopping integral, *v* represents the Coulomb interaction, and $c_{i\sigma }^{+}$ and $c_{i\sigma }$ are the Fermi creation and annihilation operators, respectively.

The s-d coupling term $H_{m-el}$ reads

(8) $$H_{m-el}=\sum_{i}I_{i}S_{i}s_{i}.$$

The parameter *I* represents the s-d(f) interaction constant, while $s_{i}$ denotes the spin operators for the conduction electrons, which can be expressed as $s_{i}^{+}=c_{i+}^{+}c_{i-}$, $s_{i}^{z}=\left(c_{i+}^{+}c_{i+}-c_{i-}^{+}c_{i-}\right)/2$.

The band gap energy is determined by the difference between the valence and conduction bands.

(9) $$E_{g}=\omega^{+}\left(k=0\right)-\omega^{-}\left(k=k_{\sigma }\right).$$

The electronic energies

(10) $$\omega^{\pm }\left(k\right)=\epsilon_{k}-\frac{\sigma }{2}I\langle S^{z}\rangle +\sum_{k^{\prime }}\left[v\left(o\right)-v\left(k-k^{\prime }\right)\right]\langle n_{k^{\prime }-\sigma }\rangle$$

are observed from the Green’s functions $g\left(k\sigma \right)=\ll c_{k\sigma };c_{k\sigma }^{+}\gg$, σ=±1. *v* is the Coulomb interaction, $\langle n_{k^{\prime }\sigma }\rangle$ is the occupation number distribution. $\langle S^{z}\rangle$ is the magnetization.

It is essential to emphasize that all quantities must be determined in a self-consistent manner.

## 3. Numerical Results and Discussion

The calculations were carried out using programs developed in the JAVA programming language. A self-consistent iterative method was employed, with the model parameters specified below serving as input for the initial iteration. For each subsequent iteration, the results from the preceding calculation were used as the input. The iterative process continued until the difference between consecutive iterations was less than a specified, sufficiently small value.

The numerical results were obtained using the following model parameters: *J*(Cr-Cr) = −3.348 meV, *D* = 0.03 meV [16], *J*(Co-Co) = −0.237 meV, *D* = 0.01 meV [17], *J* (Mn-Mn) = −1.07 meV, *D* = 0.02 meV [18], *I* = 0.2 eV, *v* = 0.3 eV, $J^{\prime }$ = 550 K, and Ω = 20 K.

It is crucial to note that ${\mathrm{Nb}}^{5+}$ is nonmagnetic, and bulk undoped KNO is also nonmagnetic. However, in KNO NPs, oxygen vacancies on the surface lead to the formation of different valence states of ${\mathrm{Nb}}^{4+}$ and/or ${\mathrm{Nb}}^{3+}$, which are paramagnetic (*S* = 1) [19,20]. This paramagnetism increases further upon ion doping. A similar enhancement in magnetization *M* has been reported for Fe-doped KNO NPs in [21].

An NP is defined by positioning the origin at a specific Nb spin located at the center of the particle and organizing all other spins into concentric shells around it. These shells are indexed by n=1,2,…,N, with n=1 corresponding to the central spin and n=N representing the surface shell. Surface effects are incorporated using distinct exchange interaction parameters $J_{s}$ for the surface layer (n=N) as opposed to the bulk parameter *J*. So, the properties are discussed on a microscopic level.

### 3.1. Size Dependence of the Magnetization *M* and Polarization *P* in KNO

**Figure 1:**
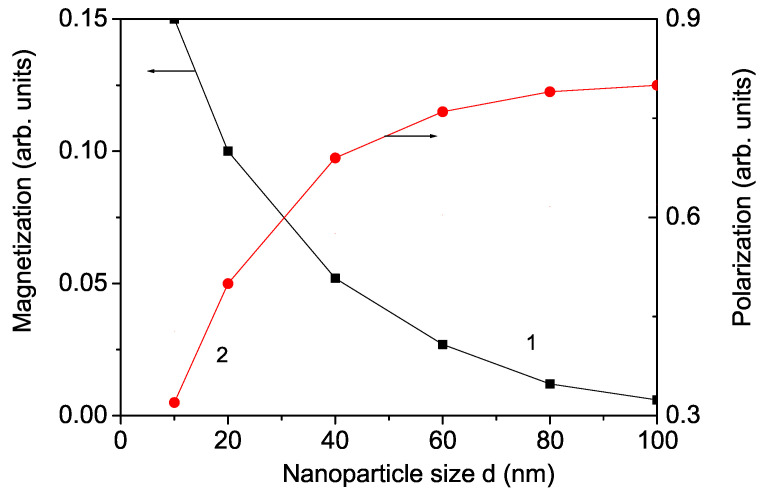
Size dependence of the magnetization *M*($J_{s}=1.2J$) (1) and polarization *P*($J_{s}^{\prime }=0.8J^{\prime }$) (2) of KNO.

Bulk undoped KNO is nonmagnetic because ${\mathrm{Nb}}^{5+}$ is nonmagnetic. But in KNO NPs, there appear oxygen vacancies on the surface causing different valence states of ${\mathrm{Nb}}^{4+}$ and/or ${\mathrm{Nb}}^{3+}$, which are paramagnetic with *S* = 1 [20]. Due to the surface oxygen vacancies, the distance between the surface ions are changed and therefore the spin exchange interaction constants on the surface $J_{s}$ or $J_{s}^{\prime }$ are tuned compared to those in the bulk *J* or $J^{\prime }$. Moreover, the oxygen vacancies cause a weak magnetization in an external magnetic field, which increases with decreasing particle size (see Figure 1, curve 1, for $J_{s}>J$). This behavior is in agreement with the experimental data of Diaz-Moreno et al. [20] for KNO NPs with an average size of 25 nm.

Due to surface effects, for the relation $J_{s}^{\prime }<J^{\prime }$, we discover that the polarization *P* decreases with decreasing NP size *d*. The result is demonstrated in Figure 1, curve 2. This is in agreement with the experimental data of Dudhe et al. [22], but in disagreement with the increase in *P* with decreasing *d* observed by Ge et al. [23] for KNO NPs and Lee et al. [24] for KNO thin films on a single crystalline Rh substrate. The Curie temperature $T_{C}$ is also reduced in KNO NPs in comparison with bulk KNO, which is attributed to surface and size effects [25].

### 3.2. Effects of Ion Doping at Rb or K Sites on Magnetization, Polarization, and Band Gap in KNO NPs

**Figure 2:**
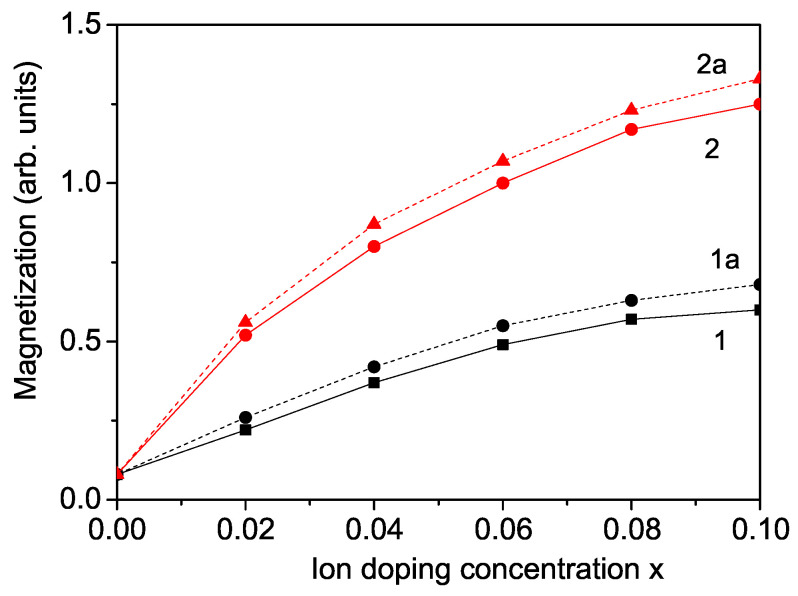
Doping concentration dependence of the magnetization *M* for a KNO NP: *d* = 20 nm for (1) Co doping at the Nb site, (2) Cr doping at the Nb site, (1a) La/Co co-doping at the K and Nb sites, (2a) La/Cr co-doping at the K and Nb sites; for *T* = 300 K, *h* = 100 Oe.

Bulk KNO is a ferroelectric material with a wide band gap ($E_{g}$ = 3.2–3.3 eV), which makes it unsuitable for visible light photocatalysis applications. However, doping KNO with transition metal ions such as Cr, Co, and Mn can lower its band gap $E_{g}$. To explore these effects, we analyzed the substitution of these ions at the Nb sites using Equations (9) and (10). While undoped bulk KNO is nonmagnetic, the introduction of transition metal ions induces a small ferromagnetism, rendering the KNO NP multiferroic. For example, doping with ${\mathrm{Cr}}^{3+}$ at the ${\mathrm{Nb}}^{5+}$ site causes compressive strain due to ${\mathrm{Cr}}^{3+}$’s smaller ionic radius (0.61 $\dot{A}$) compared to ${\mathrm{Nb}}^{5+}$ (0.69 $\dot{A}$), resulting in reduced lattice parameters. This doping also creates oxygen vacancies to balance the charge. The exchange interaction coupling $J(r_{i}-r_{j})$ is inversely proportional to the distance between spins and the lattice parameters. In the presence of compressive strain, the exchange interaction constant in the doped state, $J_{d}$, is larger than in the undoped state, $J_{d}>J$, while tensile strain results in $J_{d}<J$. Consequently, we can investigate macroscopic properties at a microscopic level. For Cr substitution, the induced compressive strain increases the exchange intraction constants in the doped states, $J_{d}>J$, leading to ferromagnetic behavior (see Figure 2, curve 2), which aligns with experimental observations by Sakthivel et al. [5]. A similar ferromagnetic behavior is seen with ${\mathrm{Co}}^{2+}$ doping in bulk KNO (see Figure 2, curve 1), as reported by Astudillo et al. [26].

**Figure 3:**
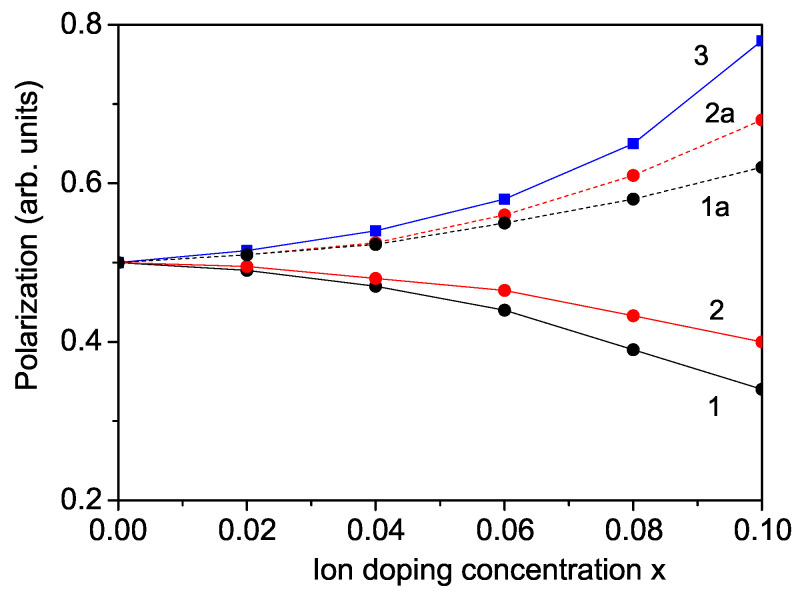
Doping concentration dependence of the polarization *P* for a KNO NP: *d* = 20 nm at room temperature for (1) Co doping at the Nb site, (2) Cr doping at the Nb site, (3) La doping at the K site, (1a) La/Co co-doping at the K and Nb sites, (2a) La/Cr co-doping at the K and Nb sites.

Upon doping KNO NPs with transition metal ions, which impart both ferromagnetic and ferroelectric properties due to magnetoelectric coupling, the polarization *P* can be also tuned. Raman spectroscopy results from Zhang et al. [9] indicate that when ${\mathrm{Co}}^{2+}$ ions substitute for ${\mathrm{Nb}}^{5+}$ in the KNO lattice, the octahedral deformation decreases the distortion along the polar axis. It is crucial to emphasize that in perovskite oxide KNO, the ferroelectric polarization *P* results from the displacement of ${\mathrm{Nb}}^{5+}$ ions from the center of symmetry within the ${\mathrm{NbO}}_{6}$ octahedron, which disrupts the spatial inversion symmetry. Consequently, the ferroelectric polarization *P* is weakened after doping with Co or other transition metal ions (Cr, Ni, Mn) at the Nb sites. Figure 3 demonstrates that the polarization *P* decreases with increasing concentrations of Cr or Co doping in bulk KNO at room temperature (*T* = 300 K). This reduction in polarization *P* for transition metal ion-doped KNO is consistent with findings by Min et al. [1], Sakthivel et al. [5], and Korde et al. [27].

**Figure 4:**
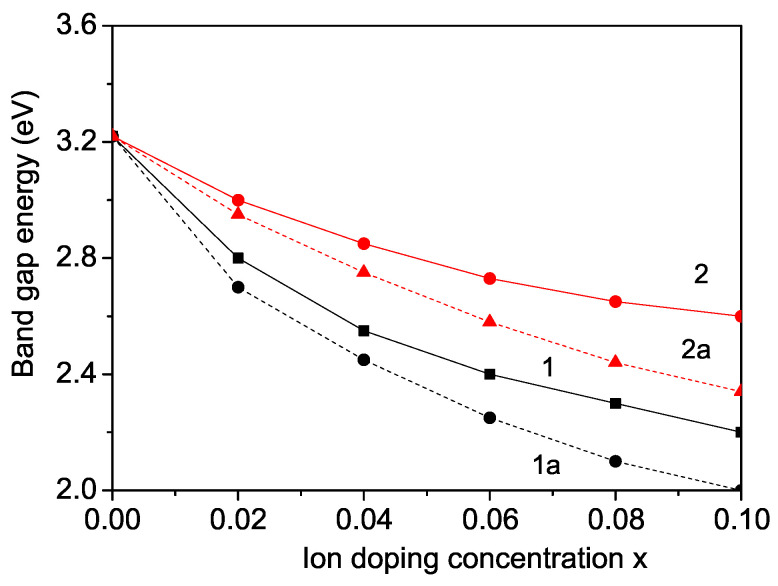
Doping concentration dependence of the band gap energy $E_{g}$ for a KNO NP: *d* = 20 nm for (1) Cr doping at the Nb site, (2) Co doping at the Nb site, (1a) La/Cr co-doping at the K and Nb sites, (2a) La/Co co-doping at the K and Nb sites at room temperature.

Doping with transition metal ions at the Nb site induces a phase transition from the orthorhombic to the cubic phase in KNO NPs and leads to a significant reduction in the optical band gap. According to Equations (9) and (10), an increase in magnetization $\langle S^{z}\rangle$ with higher doping concentrations x (see Figure 2) corresponds to a decrease in the band gap energy $E_{g}$, as illustrated in Figure 4, curves 1 and 2. This phenomenon has been experimentally observed in Cr- and Co-doped KNO by Sakthivel et al. [5] and Zhang et al. [9]. Therefore, the altered band gap of doped KNO NPs holds potential for applications in solar photovoltaic technologies.

To the end of this section, we will examine the effects of substituting K ions with Ba, La, Na, or Li ions. The ionic radii of Ba (1.49 $\dot{A}$), La (1.17 $\dot{A}$), Na (1.16 $\dot{A}$), and Li (0.90 $\dot{A}$) ions are smaller than that of the $K^{+}$ ion (1.52 $\dot{A}$), resulting in a compressive strain. This compressive strain enhances the pseudo-spin exchange interaction constant $J_{d}^{\prime }$ (where $J_{d}^{\prime }>J^{\prime }$) and increases the polarization *P* with higher doping concentrations *x* compared to the undoped material. We calculated the polarization for La-doped KNO NPs at the K site at room temperature, as shown in Figure 3, curve 3. A similar enhancement in electric properties for Na- and Li-doped KNO has been reported by Wang et al. [28] and Trepakov et al. [29].

### 3.3. Co-Doping Effects with Different Ions at Both K and Nb Sites on Various Properties of KNO NPs

Our goal was to reduce the band gap energy $E_{g}$ in KNO NPs through ion doping. However, doping transition metal ions at the Nb site results in a reduction in the polarization *P* (see Figure 3, curves 1 and 2). Conversely, doping with ions such as La, Ba, Na, or Li at the K site increases the polarization but does not decrease the band gap energy. To address this issue, we propose co-doping with different ions at both K and Nb sites. This approach will be investigated for the first time using the proposed microscopic model and Green’s function technique.

First, we will study the co-doping La/Co and La/Cr effects on the magnetization *M*. We observe an enhanced *M* in co-doped KNO NPs, mainly due to the substituted transition metal ions Co or Cr, as reported in [4,30]. The results are presented in Figure 2 with curves 1a and 2a. The co-doped KNO materials present room-temperature ferromagnetism.

The increased magnetization leads to a reduction in the band gap energy $E_{g}$, as demonstrated in Figure 4, curves 1a and 2a. Co-doping KNO with different ions results in a more pronounced decrease in $E_{g}$ compared to doping solely at the Nb site. For La/Cr co-doped KNO, the band gap can be tuned from 3.22 eV to 2.0 eV (curve 1a), while for La/Co co-doped KNO, it ranges from 3.22 eV to 2.34 eV (curve 2a). This outcome aligns well with experimental results for La/Co co-doped KNO, as reported by Zhang et al. [9], who observed a reduction from 3.22 eV to 2.31 eV for xxx ranging from 0 to 0.12. This agreement supports the validity of our model and methodology in explaining the properties of co-doped KNO. Similar reductions in band gap energy are also observed for other co-doped KNO systems, such as Ba/Fe (from 3.25 eV to 1.72 eV, *x* = 0–1.0 [30]), Ba/Co (from 3.26 eV to 1.87 eV, *x* = 0–0.75 [31]), and Ba/Ni (from 3.30 eV to 1.84 eV, *x* = 0.3 [8], and down to 1.39 eV, *x* = 0.1 for KNO thin films [32].

Let us examine the polarization *P* in La/Co and La/Cr co-doped KNO NPs. As illustrated in Figure 3, curves 1a and 2a, the overall effect of co-doping is an increase in polarization *P* with higher doping concentrations *x* for both La/Co and La/Cr. Although the polarization *P* in these co-doped NPs is slightly lower than that observed with La ions substituted at the K site, it remains higher than in undoped KNO. A similar trend is observed for magnetization *M*, polarization *P*, and band gap energy $E_{g}$ in co-doped KNO systems such as Ba/Co or Ba/Cr. Enhanced ferroelectric properties have been reported in various co-doped KNO materials, including La/Co, Ba/Co, and Ba/Ni co-doped KNO [4,9,31].

## 4. Conclusions

In summary, we have employed a microscopic model and Green’s function theory to explore the effects of co-doping on ferroelectric KNO NPs. We have studied the co-doping ions La/Cr and La/Co, which leads to increasing the magnetization *M* and polarization *P*, as well as to strongly decreasing the band gap energy $E_{g}$. The transition metal ions Cr and Co, which substitute the Nb ions, contribute mainly to the tuning of the magnetization *M* and band gap energy $E_{g}$, whereas the La or Ba ions, which substitute the K host ions, lead to raising the polarization *P*, which is reduced by the transition metal ions. Substitution with two or more ions allows for more effective and flexible modification of the different properties of KNO NPs, making them suitable for diverse applications. Let us emphasize that the magnetization in the co-doped KNO NP is larger than that in the bulk, $M{\left(x\right)}^{NP}>M{\left(x\right)}^{b}$, whereas via polarization, it is vice versa, $P{\left(x\right)}^{NP}<P{\left(x\right)}^{b}$. The co-doped KNO materials exhibit multiferroic characteristics with a reduced band gap energy, room-temperature ferromagnetism, and high ferroelectricity, all of which can be adjusted through doping levels. These findings are in good qualitative agreement with experimental data. Therefore, co-doped KNO—both in bulk form and as NPs—shows potential for use in multiferroic devices and solar photovoltaic applications.

## Data Availability

Derived data supporting the findings of this study are available from the corresponding author upon reasonable request.
